# Obituary

**Published:** 1897-07

**Authors:** 


### Obituary.

Died at his home in Keokuk, Ia., May 24th, 1897, Dr. L. C. Ingersoll, aged 73 years, 7 months and 28 days.

Dr. Ingersoll was a native of New York; he took up his residence in Keokuk in 1858 and has resided there continuously since that time.

Dr. Ingersoll's professional life was a very active one, he having a warm interest in all that pertained to the welfare of his chosen profession. Indeed, he was a leader. He was ever interested and active in professional societies, and especially that of his own State, of which he was president for several years.

He did much to further the interests of association work in his profession. He was a member of the faculty of the Dental Department of the Iowa State University for ten years and during all that time was its Dean.

He was for twenty-five years a teacher in medical and dental colleges. He was a member of the faculty of the Keokuk Medical College from its organization.

He added much to the literature of the profession. He was the author of a work on dental pathology which was highly appreciated in the profession; he added much to dental journalism.

He was a man of high scholarly attainments, was of a kind and gentle disposition, was generous and helpful and an inspiration to all who knew him.

The removal by death of such men as Dr. Ingersoll, produces a chasm that is not readily filled.

Dr. Ingersoll was twice married. His first wife was Miss Maria Porter, who died in 1888. His second wife was Miss Minnie Banks to whom he was married in 1890 and survives him.

The following resolutions of respect for Dr. Ingersoll, were unanimously passed at a meeting of the dentists of Keokuk:

WHEREAS, The Allwise Ruler of the Universe has, in his infinite wisdom, removed from our midst our highly esteemed fellow-laborer, Luman C. Ingersoll, and

WHEREAS, The intimate relation held by him during a long and useful life with the dentists of Keokuk, Ia., makes it fitting that we record our appreciation of him; therefore

*Resolved*, That the wisdom, fidelity and ability which he has exercised in science, literature, art, research and council to aid in establishing and advancing the profession of his choice, will always be held in grateful remembrance:

*Resolved*, That the removal of such a man from among us where he has held a leading position for more than forty years, leaves a vacancy that will be deeply realized by all.

*Resolved*, That we extend to his bereaved family and relatives our earnest sympathy and mourn with them in this sad affliction.

D. W. Mills,
B. C. Hinkley,
J. W. Stark,
*Committee.*

